# Reverse Shoulder Arthroplasty Baseplate Stability Is Affected by Bone Density and the Type and Amount of Augmentation

**DOI:** 10.3390/bioengineering12010042

**Published:** 2025-01-08

**Authors:** Daniel Ritter, Patric Raiss, Patrick J. Denard, Brian C. Werner, Manuel Kistler, Celina Lesnicar, Micheal van der Merwe, Peter E. Müller, Matthias Woiczinski, Coen A. Wijdicks, Samuel Bachmaier

**Affiliations:** 1 Department of Orthopedic Research, Arthrex, 81249 Munich, Germany; 2 Department of Orthopaedics and Trauma Surgery, Musculoskeletal University Center Munich (MUM), University Hospital, LMU Munich, 80336 Munich, Germany; 3 OCM Clinic, 81369 Munich, Germany; 4 Southern Oregon Orthopedics, Medford, OR 97504, USA; 5 Department of Orthopaedic Surgery, University of Virginia Health System, Charlottesville, VA 22908, USA; 6 Experimental Orthopaedics University Hospital Jena, Campus Eisenberg, Friedrich-Schiller-University, 07607 Eisenberg, Germany

**Keywords:** reverse shoulder arthroplasty, preoperative bone density, CT imaging, augment, baseplate, primary fixation, BIO-RSA

## Abstract

Objective: This study evaluated the effects of bony increased offset (BIO) and metallic augments (MAs) on primary reverse shoulder arthroplasty (RSA) baseplate stability in cadaveric specimens with variable bone densities. Methods: Thirty cadaveric specimens were analyzed in an imaging and biomechanical investigation. Computed tomography (CT) scans allowed for preoperative RSA planning and bone density analysis. Three correction methods of the glenoid were used: (1) corrective reaming with a standard baseplate, which served as the reference group (*n* = 10); (2) MA-RSA (*n* = 10); and (3) angled BIO-RSA (*n* = 10). Each augment group consisted of 10° (*n* = 5) and 20° (*n* = 5) corrections. Biomechanical testing included cyclic loading in an articulating setup, with optical pre- and post-cyclic micromotion measurements in a rocking horse setup. Results: There were no differences in bone density between groups based on CT scans (*p* > 0.126). The BIO-RSA group had higher variability in micromotion compared to the MA-RSA and reference groups (*p* = 0.013), and increased total micromotion compared to the reference group (*p* = 0.039). Both augmentations using 20° corrections had increased variance in rotational stability compared to the reference group (*p* = 0.043). Micromotion correlated with the subchondral bone density in the BIO-RSA group (r = −0.63, *p* = 0.036), but not in the MA-RSA (*p* > 0.178) or reference (*p* > 0.117) groups. Conclusions: Time-zero baseplate implant fixation is more variable with BIO-RSA and correlates with bone density. Corrections of 20° with either augmentation approach increase variability in rotational micromotion. The preoperative quantification of bone density may be useful before utilizing 20° of correction, especially when adding a bone graft in BIO-RSAs.

## 1. Introduction

Reverse shoulder arthroplasty (RSA) is an effective treatment for shoulder pathologies, including osteoarthritis and rotator cuff deficiency [1]. The first RSA designs medialized the center of rotation (COR). However, high rates of scapular notching and limited rotational range of motion led to implant modifications. One of the major adjustments was glenoid-sided lateralization. Shifting the COR laterally compared to earlier designs lowered the risk of bony impingement [2,3,4]. However, lateralization increases stress at the baseplate–glenoid interface.

From a technical perspective, it is advised to avoid a superior tilt of the glenoid baseplate to maximize stability. Given the concavity of the glenoid, correction of the RSA angle is necessary to achieve appropriate baseplate contact. While inferior reaming is an option, this approach can require extensive bone removal and lead to medialization. Alternatively, correction of the RSA angle with the maintenance of bone stock and net lateralization can be achieved with either bone grafts (bony increased offset (BIO)) or metal augments (MAs) [1,5,6,7,8,9]. Notably, increased lateralization in combination with poor bone density may compromise primary implant fixation [10,11,12] and increase complications, with significant effects on long-term patient outcomes [13,14,15,16,17]. Prior studies have suggested that computed tomography (CT) scans used for preoperative planning may provide valuable information about bone density [18,19,20]. Biomechanical data comparing baseplate stability and micromotion with BIO- or MA-RSA are lacking [21,22,23]. The effects of bone density and amount of RSA angle correction on time-zero baseplate fixation can improve preoperative data to optimize surgical decision-making.

The purpose of this study was to compare the time-zero implant micromotion of BIO-RSA and MA-RSA with different amounts of inclination correction in relation to preoperatively analyzable glenoid bone density. It was hypothesized that BIO-RSAs would have decreased time-zero stability compared to MA-RSAs, particularly with increased correction and lower bone density.

## 2. Materials and Methods

**Figure 1:**
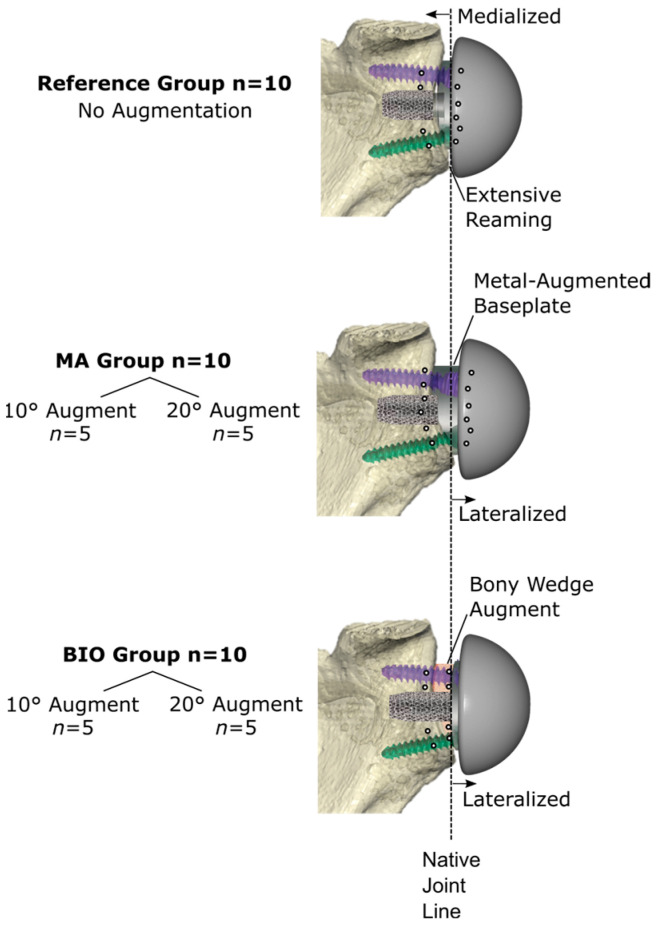
Group assignment of the specimen based on virtually planned reverse shoulder arthroplasty. Bone density analysis and biomechanical testing for the reference, metal augment (MA) and bony increased offset (BIO) groups, which consisted of (*n* = 5) 10° and 20° augmentation, respectively. The compression screws in the inferior position are shown by green screws with locking screws (purple) in the superior position.

A biomechanical investigation was performed on 30 cadaveric shoulder specimens. All specimens (mean age 71.9 ± 11.9 years; 13 females and 17 males) were scanned in a clinical CT scanner for surgical planning, followed by bone density and glenoid morphology assessments. Specimens were assigned into three groups, including two treatment groups with augmentation and one reference group without augmentation. The treatment groups included *n* = 10 MAs and *n* = 10 angled BIO-RSAs. Preoperative planning allowed the assignment of five specimens with 10° correction and five specimens with 20° correction to each treatment group (Figure 1).

### 2.1. Bone Density

Bone density parameters were extracted from scans performed in a standard clinical CT scanner (Siemens SOMATOM Definition AS+, Siemens Healthcare GmbH, Erlangen, Germany) with a voxel resolution of 0.6 × 0.6 × 0.6 mm. Patient-specific calibration was performed according to previous studies to make this bone density analysis applicable to multicentric standard preoperative CT data [18,19,24]. Grayscale values were converted into bone mineral density (BMD) values by linearly interpolating grayscale values on defined BMD air fat and muscle values [−840, −80 and 30 mgHA/cm^3^]. The morphological parameter (BV/TV) calculation of each VOI was based on pixel-counting methods using the respective bone volume (BV) of the total volume (TV).

#### Regions of Interest

**Figure 2:**
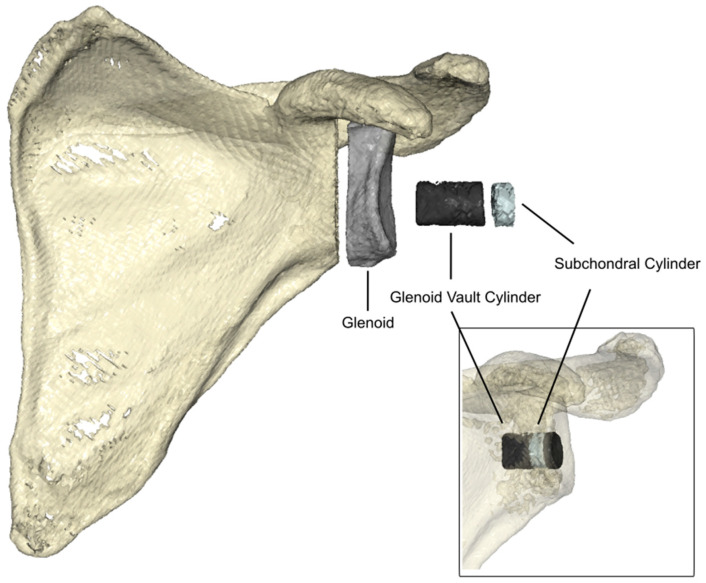
Evaluation of the bone density in the respective regions of interest: glenoid, cylindrical glenoid vault, and subchondral cylinder, demonstrated in three three-dimensionally rendered CT images.

Global volumes of interest (VOIs) included the glenoid bone portion (Figure 2). Regions of interest were evaluated along the scapular axis, through the root of the scapular spine and the middle point of the glenoid. Regions of interest relevant for implant stability included a subchondral and glenoid vault cylinder (Figure 2). The diameter of both cylinders was defined as 50% of the 3 to 9 o’clock glenoid distance measured at the articular surface. The depth of the glenoid vault cylinder was defined as up until one endpoint of the cylinder reached the medial cortex. The subchondral cylinder depth was determined using one-third of the glenoid vault cylinder, starting medially to the articulating cortex below necrotic bone tissue. All regions of interest were cropped to use the pixel information, using a global segmentation threshold for consistent bone density extraction (Figure 2).

### 2.2. Virtual Planning and Surgical Technique

Specimens (Science Care Inc., Phoenix, AZ, USA) were stored at −20 °C and thawed at room temperature for at least 24 h before tissue preparation and testing. CT imaging-based preoperative planning was performed using a commercially available three-dimensional preoperative planning software (Virtual Implant Positioning version 8.1.0, Arthrex; Naples, FL, USA). A 24 mm circular baseplate (Modular Glenoid System; Arthrex; Naples, FL, USA) was positioned 3 mm superior to the inferior glenoid rim in all specimens. Implantation was planned to achieve 5° of implant retroversion and a 0° RSA angle. This defined the degree (10° or 20°) of full-wedge augmentation. The correct wedge orientation was achieved by slightly rotating the implant around its axis to reach the minimum baseplate or bone graft contact area threshold of 80%. Extensive bone reaming was necessary for the non-augmented reference group to provide 0° inclination and 5° retroversion. Minimal reaming was performed in the glenoids planned for either BIO- or MA-RSA augmentation. All cases (*n* = 30) were planned and corrected to a 0° RSA angle and 5° retroversion with the augment that demonstrated the least amount of reaming. The specimens were then assigned into the following groups: 10° BIO (*n* = 5), 10° MA (*n* = 5), 20° BIO (*n* = 5), 20° MA (*n* = 5), and Reference (*n* = 10). The bone and metal augmentations were planned in the same manner, as a bone grafting instrument set allowed for the reproducible preparation of the respective grafts.

All the baseplates were placed using a reusable patient-specific guide (Glenoid Targeter; Arthrex, Naples, FL, USA) to ensure replication of the planned position. The central baseplate post and peripheral screws were implanted in a bicortical fashion using the respective screw and post length. The post length anchoring in the native glenoid was consistent for all groups (BIO: 26 ± 4 mm; MA: 24 ± 5 mm; Reference: 26 ± 4 mm). The screws consisted of a non-locking compression screw in the inferior position and three more locking screws, which were kept consistent for all groups. Standard glenospheres were used without additional lateralization or inferiorization, and the diameter was defined according to the plan to achieve a 3 mm inferior overhang. The amount of lateral offset was defined by the degree of augmentation. The 10° and 20° augmentations resulted in an additional 2.1 mm and 4.4 mm of lateral offset, respectively (Figure 1). To achieve the 0° inclination and 5° retroversion in the reference group, additional glenoid reaming resulted in 2.1 to 4.4 mm medialization of the COR in reference to the native joint line [25,26].

### 2.3. Biomechanical Testing

**Figure 3:**
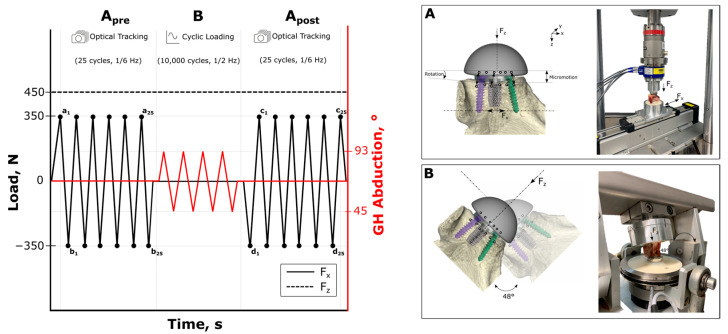
Test protocol and phases of the two test setups (**left**): phase (**A**): shear (Fx) and compressive loading (Fz), with definition of the measurement of outcome variables before (pre, mean Δa_1_b_1_ to Δa_25_b_25_) and after (post, mean Δc_1_d_1_ to Δc_25_d_25_) fatigue testing; phase (**B**): fatigue testing with cyclic abduction under compressive loading (Fz) in two experimental setups (**right**), setup A: biaxial rocking-horse setup for shear and compressive loading during micromotion detection, and setup B: articulation simulator for implant loosening.

Biomechanical testing was developed in accordance with the ASTM F2028-17 testing standard [27]. The testing procedure was conducted in two phases, in two custom test setups (Figure 3). Shear and compression loads were applied in a biaxial rocking-horse setup, while measuring the displacement of the glenoid baseplate (setup A). Cyclic rotation of the glenoid component was performed in an articulation simulator (setup B) to simulate shoulder abduction. Following the articular cyclic loading, the displacement of the glenoid baseplate was recorded again in setup A using the same shear and compressive loading setup. All specimens were embedded centrally in an aluminum pot using polyurethane resin (RenCast^®^ FC 52/53 Isocyanate and FC 53 Polyol, Huntsman Advanced Materials (Europe) BV, Everberg, Belgium). The articulating surface of the glenosphere component was carefully aligned horizontally, and the embedding material was filled to a level at a safe distance from the implant or screws.

For setup A, the specimens were mounted on the biaxial testing setup (Figure 3). The load was applied via a humeral polyethylene component matching the glenosphere size, fixed in a universal testing machine (Instron ElectroPuls E10000, Norwood, MA, USA) with a six-component load cell (MCS10-010-6C, Baldwin Messtechnik GmbH, Darmstadt, Germany). A constant compressive load of 430 N was applied through the COR of the joint, perpendicular to the glenoid plane, while the specimens were moved cyclically along the superior–inferior axis, parallel to the glenoid plane, using a linear actuator (RK DuoLine S 80, Phoenix Mecano, Stein am Rhein, Switzerland) for a reproducible shear force application of 350 N, with a frequency of 1/6 Hz, for 25 load cycles (Figure 3). In accordance with the ASTM standard, the measuring frequency did not exceed 200 N/s.

In setup B, the embedded specimens were clamped into the articulation simulator (Figure 3). All the reverse glenoid components were cyclically rotated from 45° to 93° (a total of 48°) of abduction around the matching humeral polyethylene component. A total of 10,000 load cycles, with a compressive load of 430 N, were applied, at a frequency of 0.5 Hz.

### 2.4. Outcome Variables

Primary stability was defined as micromotion at the implant–glenoid interface (Figure 3). To assess the primary stability of the glenoid implants, the spatial displacement of the glenoid component was recorded pre- and post-cyclically during the rocking-horse-tests (setup A) using an optical measuring system (Aramis, 3D Camera 2.3M, Carl Zeiss GOM Metrology, Oberkochen, Germany), with a frequency of 5 Hz for 25 cycles. The camera configuration ensured a measuring volume of 140 mm × 90 mm × 90 mm and a measuring accuracy of 2.8 μm within and 5.6 μm outside of the focus plane. For motion detection, six measuring points were fixed to the surface of the glenosphere and the glenoid rim, and, if applicable, to the bony augment (Figure 3). Micromotion was calculated as the relative displacement between the glenoid rim and the glenosphere. Rotational displacement resulted from two lines extending from the first to the last measuring point on the glenosphere and the glenoid, respectively. Rotational displacement and micromotion were analyzed as the mean minimum to maximum values of each shear load cycle before (pre, mean Δa_1_b_1_ to Δa_25_b_25_) and after (post, mean Δc_1_d_1_ to Δc_25_d_25_) fatigue testing (Figure 3). For the BIO group, the bone graft was separately tracked optically to quantify the percentages of graft–glenoid and implant–graft micromotion, respectively. The outcome data of the implant–bone interface analysis included pre- and post-cyclic rotation and micromotion and the respective evaluated pre-to-post-cyclic differences.

### 2.5. Statistical Analysis

The biomechanical testing outcome metrics were the dependent primary outcome variables. Bone density variables were defined as secondary outcome variables. Correlation analysis was performed between primary and secondary outcome variables using Pearson’s correlation coefficients. Statistical analysis was performed using commercial software (Sigma Plot Statistics for Windows, version 13.0, Systat Software, San Jose, CA, USA).

The statistical analysis comprised a one-way ANOVA with a Holm–Sidak post hoc test, conducted for a significant pairwise analysis of the primary outcome variables. Significance was defined as *p* ≤ 0.05. The observed post hoc average power value of all the one-way ANOVA tests exceeded the desired power level of 0.8, confirming that the sample size was sufficient. No prior sample size calculation was performed, as no matching mean and standard deviation values were found for our outcome variables and methods. Shapiro–Wilks and Brown–Forsythe tests confirmed that each data set adhered to a normal distribution and equal variance, respectively. For data sets that failed these tests, a non-parametric test (Kruskal–Wallis) was used for non-normal-distributed data sets. For Kruskal–Wallis tests that found significance, Dunn’s post hoc tests, including Bonferroni correction, were conducted to further analyze the differences.

## 3. Results

**Figure 4:**
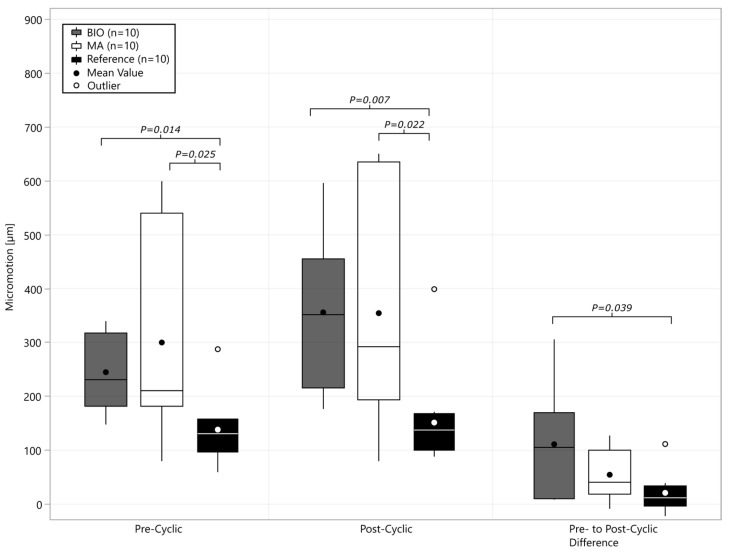
Boxplot with mean and median overall cyclic micromotion before (pre-) and after (post-) cyclic loading. Micromotion added through cyclic loading is quantified as pre- to post-cyclic difference.

**Figure 5:**
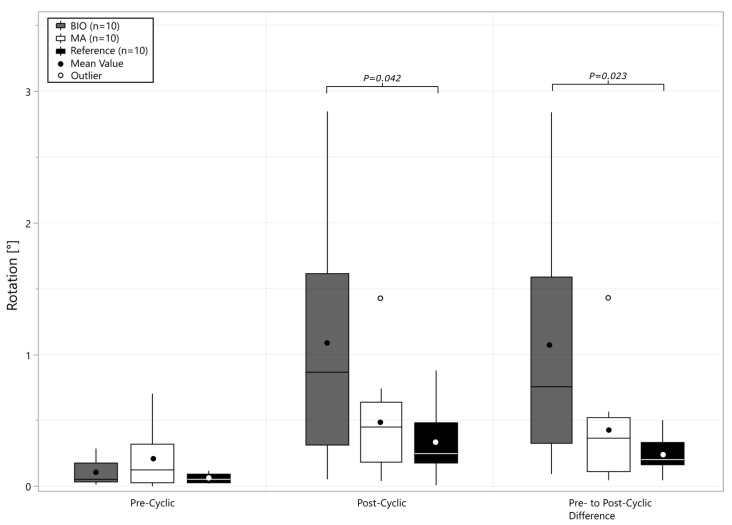
Boxplot with mean and median overall rotational displacement before (pre-) and after (post-) cyclic loading, and quantified difference. Rotation added through cyclic loading is quantified as pre- to post-cyclic difference.

**Figure 6:**
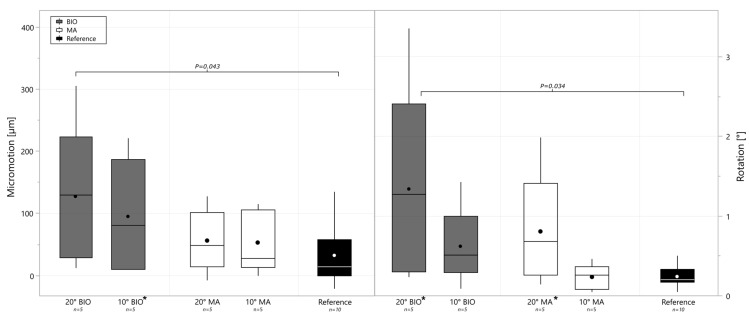
Boxplot with mean and median difference, quantifying micromotion from pre- to post-cyclic change in micromotion (**left**) and rotational displacement (**right**), divided per degree and type of augmentation. Group names marked with asterisks showed significantly increased variance compared to reference group.

There were no differences in bone density (Table 1) between the two treatment and reference groups for comparisons in Figure 4 and Figure 5 or the groups assigned based on type and amount of augmentation (*p* > 0.126), as shown in Figure 6.

BIO baseplate fixation resulted in a higher variability in micromotion compared to the MA-RSA and reference groups (*p* = 0.013), and increased total micromotion (*p* = 0.039) compared to the reference group (Figure 4). The micromotion in the BIO-RSA group had a similar percentage of displacement at the implant–graft (53 ± 14%) and the graft–glenoid interfaces (47 ± 16%), resulting in similar micromotion to that of the reference and MA groups. Micromotion in the MA-RSA (*p* > 0.178) and reference (*p* > 0.117) groups did not correlate significantly with bone density, while a significant correlation with subchondral bone density (cylindrical BV/TV) was found for the BIO group (r = −0.63, *p* = 0.036).

In the pre-/post-cyclic comparison (Figure 5) of the rotational displacement, the BIO-RSA group had higher variability than the MA and reference groups (*p* < 0.033), and increased micromotion compared to the reference group (Figure 5, *p* = 0.023).

As is visible in Figure 6, both augmentation approaches resulted in significantly higher rotational fixation variability (*p* = 0.034) when using 20° of correction. The 10° BIO-RSA correction group had increased variance in micromotion compared to the reference group (*p* = 0.043), and the 20° BIO-RSA group had significantly higher micromotion and rotational displacement than the reference group (Figure 6).

## 4. Discussion

The most important findings of this study are that time-zero implant–bone micromotion of an RSA glenoid baseplate is affected by (1) the method of angular correction (bone versus metal), (2) the amount of angular correction, and (3) variability in bone density. BIO-RSA baseplate stability was more variable with increased micromotion and rotational displacement. Implant micromotion when using angled BIO-RSA was also correlated with glenoid subchondral bone density, with a higher degree of RSA angle correction (20°) resulting in increased variability in rotational stability compared to MAs. While the impact of micromotion on bone resorption and clinical outcomes remains to be determined, assessment of glenoid bone density with preoperative CTs may be a helpful tool to screen the bone density for informed decision-making regarding metal or bone as a means of angular deformity correction during RSA.

The long-term bone graft behavior of BIO-RSA procedures may be related to the mechanical environment, and there is a risk of graft resorption, which may influence clinical outcomes due to implant loosening and scapular notching [9,28,29]. The clinical importance of graft fixation is currently undergoing discussion, as the 5-year survival rate is reliable, and anchoring the baseplate without a graft behind has also been reported to have positive outcomes (the “stilting” technique) [30]. However, it can be difficult to precisely control the intended bony wedge size due to both graft preparation and potential compression during implantation. Experienced surgeons may not be challenged by preparing and implanting the graft reproducibly; however, MAs reduce the graft variables and achieve the desired contact area through glenoid preparation.

Considering the micromotion at the interfaces of the BIO-RSA constructs separately (the glenoid–graft and implant–graft interfaces), the respective micromotions were in a similar range as for the MA and reference groups, which may indicate clinically reliable healing rates with BIO-RSA as well as with MAs [31,32]. However, the significant correlation with bone density in the BIO-RSA group and the addition of an interface between the bony augment and the baseplate explain the higher variability and the overall increase in micromotion. Undefined bone graft compression due to varying fixation techniques and bone densities may be reasons for the clinical appearances of stress shielding, implant loosening, and scapular notching [9,28,29]. Thus, MAs may be preferred, particularly in patients with poor bone quality, as less variable fixation stability when using these implants did not correlate with the bone density. Generally, high degrees of lateralization or large augment angles should be applied carefully in patients with poor bone densities to achieve adequate primary fixation. Notably, higher fixation variability was found in our study when using 20° augments, regardless of the bone density and augment type utilized.

In revision scenarios or highly retroverted and inclined glenoids, healed autologous bone grafts enable bone preservation or even bone formation in patients needing long-term or revisable joint reconstructions. The in vivo biological effects on autologous bone healing are unclear, and cannot be reproduced biomechanically; however, this study showed significant differences between current implant types according to varying bone densities. The CT-based planning and investigation of primary fixation for variable bone densities may benefit the surgeon in terms of implant selection and fixation. The proposed bone density evaluation and applicability in clinical CT scans allows objective preoperative bone density classification, with a significant impact on time-zero implant stability, treatment, and stress shielding in RSA patients [17,24]. Biomechanical studies on primary augmented baseplate stability are rare, and focus on ex vivo models in artificial bone or finite element analyses. The results from previous studies are similar to our findings, with increased stresses in bony augments and higher degrees of lateralization [21,22,23]. Our study bridges the gap to the clinical situation by using standard clinical preoperative CT scans of cadaveric specimens and an articulating loading setup. The results in an artificial bone substitute are viable as confounders can be reduced, but they significantly deviate from measurements in cadaveric bone [23]. The subject-specific evaluation of preoperative plans for respective treatments, including bone density, therefore increase the clinical relevance of our study. The articulating test setup used in this study represents the most similar conditions to an in vivo situation available in the current literature. Rehabilitation-relevant load levels and standardized pre- and post-cyclic micromotion measurements allowed us to analyze the effects of in vivo-like cyclic loading on implant micromotion. Micromotion did not exceed the 150 µm limit for osseointegration [10,11,12] in the pre- to post-cyclic observations, except for in BIO-RSA constructs with poor bone densities, which exceeded this value slightly. In a clinical use case, patients below a specified bone density threshold could be detected, who are at risk for greater micromotions. Adaption of the surgical technique to reduce undefined graft compression states could be a consequence of preoperative risk stratification. For example, machine learning models could be used for clearer detection to give suitable recommendations for patients with poor bone density [18,19].

Other factors may influence the fixation of the baseplate and the behavior of an augment [33]. One biomechanical study recommended that the surgical technique may be modified according to the patient’s bone density and necrotic bone regions [34]. Constant refinement of preoperative evaluation models by adding and optimizing parameters is necessary to handle the multifactorial nature of shoulder reconstructions and their complications. The patient-specific calibration method in our study helped to reduce inaccuracies due to intra- and interscanner variations, and to make bone density analyses applicable in a multicentric context [35,36]. The concept proposed in this study, using systematically mapped bone densities in the glenoid, demonstrates promising value for its inclusion in CT-based preoperative planning, but it may still need to be validated for everyday clinical application [37]. While aseptic glenoid loosening is rare, complication rates remain increased in patients with poor bone density [17]. With an aging population and the common performance of glenoid lateralization in RSA, the preoperative identification of patients with low bone density may have implications for treatment approaches. The implementing implantation-relevant bone density parameters (subchondral (below potential necrosis) and glenoid vault cylinder) showed the highest level of interaction with implant stability in the BIO-RSA procedure. Both treatments remain valid options, but preoperative bone quality assessment may pay off by offering better preoperative knowledge in surgeons’ decision processes and potentially improving patient outcomes [20,38,39].

There are several limitations to the current study that are important to acknowledge. Bony ingrowth, which provides secondary anchorage in clinical applications, could not be accounted for in the cadaveric specimens. The in vivo stability and the load transfer of the investigated implants may behave differently over a more extended follow-up period. The biological effects and the ability of different implant types and designs to promote bone ingrowth are pertinent questions beyond this study’s scope. Nevertheless, the findings of this work determine the primary stability of BIO- and MA-RSA before bone ingrowth, and provide insight into primary implant behavior in an experimental biomechanical study. Cyclic loading in an articulating setup allowed for the simulation of glenohumeral abduction, but did not address variable in vivo shoulder joint loading and variable rotational and shear forces. Our findings are limited to a modular glenoid baseplate using standardized augmentation; multiplanar and severe defects should be researched in future studies. Patient-specific calibration may not have been necessary for this study, as all the specimens were scanned in one scanner with the same settings, but it may help in applying the bone density model multicentrically [40]. The comparison of multicentric scans and their subsequent validation with density phantoms represent future research questions.

## 5. Conclusions

Time-zero baseplate implant fixation is more variable with BIO-RSA and correlates with bone density. Corrections of 20° with either augmentation approach increase variability in rotational micromotion. The preoperative quantification of bone density may be useful before utilizing 20° of correction, especially when adding a bone graft in BIO-RSAs.

## Figures and Tables

**Table 1:** Mean values with standard deviations of density variables (BMD—bone mineral density; BV/TV—bone volume/total volume) for specimens assigned to reference, metal augment (MA), and bony increased offset (BIO) groups, including statistical analysis.

Bone Density Variables	MA	BIO	Reference	MA vs. BIO *p*-Value	MA vs. Ref. *p*-Value	BIO vs. Ref. *p*-Value
Subchondral Cylinder BMD [mgHA/cm^3^]	217 ± 34	211 ± 16	238 ± 37	0.905	0.256	0.121
Glenoid Cylinder [mgHA/cm^3^]	409 ± 49	385 ± 32	397 ± 57	0.515	0.837	0.852
Glenoid Global [mgHA/cm^3^]	371 ± 58	347 ± 30	386 ± 35	0.447	0.697	0.121
Subchondral Cylinder BV/TV	0.62 ± 0.15	0.59 ± 0.18	0.66 ± 0.14	0.906	0.820	0.568
Glenoid Cylinder BV/TV	0.72 ± 0.13	0.69 ± 0.11	0.73 ± 0.12	0.811	0.987	0.722
Glenoid Global BV/TV	0.78 ± 0.13	0.73 ± 0.11	0.80 ± 0.10	0.548	0.976	0.426

## Data Availability

Dataset available on request from the authors.

## References

[1] Bacle G., Nové-Josserand L., Garaud P., Walch G. (2017). Long-Term Outcomes of Reverse Total Shoulder Arthroplasty: A Follow-up of a Previous Study. J. Bone Joint Surg. Am..

[2] Berhouet J., Garaud P., Favard L. (2014). Evaluation of the role of glenosphere design and humeral component retroversion in avoiding scapular notching during reverse shoulder arthroplasty. J. Shoulder Elbow Surg..

[3] Levigne C., Boileau P., Favard L., Garaud P., Mole D., Sirveaux F., Walch G. (2008). Scapular notching in reverse shoulder arthroplasty. J. Shoulder Elb. Surg..

[4] Werthel J.D., Walch G., Vegehan E., Deransart P., Sanchez-Sotelo J., Valenti P. (2019). Lateralization in reverse shoulder arthroplasty: A descriptive analysis of different implants in current practice. Int. Orthop..

[5] Boileau P., Moineau G., Roussanne Y., O’Shea K. (2011). Bony Increased-offset Reversed Shoulder Arthroplasty: Minimizing Scapular Impingement While Maximizing Glenoid Fixation. Clin. Orthop. Relat. Res.^®^.

[6] Boileau P., Morin-Salvo N., Bessière C., Chelli M., Gauci M.-O., Lemmex D.B. (2020). Bony increased-offset–reverse shoulder arthroplasty: 5 to 10 years’ follow-up. J. Shoulder Elb. Surg..

[7] Dimock R., Fathi Elabd M., Imam M., Middleton M., Godenèche A., Ali Narvani A. (2021). Bony increased-offset reverse shoulder arthroplasty: A meta-analysis of the available evidence. Shoulder Elb..

[8] Imiolczyk J.-P., Audigé L., Harzbecker V., Moroder P., Scheibel M. (2022). Metallic humeral and glenoid lateralized implants in reverse shoulder arthroplasty for cuff tear arthropathy and primary osteoarthritis. JSES Int..

[9] Merolla G., Giorgini A., Bonfatti R., Micheloni G.M., Negri A., Catani F., Tarallo L., Paladini P., Porcellini G. (2023). BIO-RSA vs. metal-augmented baseplate in shoulder osteoarthritis with multiplanar glenoid deformity: A comparative study of radiographic findings and patient outcomes. J. Shoulder Elb. Surg..

[10] Favre P., Seebeck J., Thistlethwaite P.A., Obrist M., Steffens J.G., Hopkins A.R., Hulme P.A. (2016). In vitro initial stability of a stemless humeral implant. Clin. Biomech..

[11] Peppers T. (1998). Fixation of humeral prostheses and axial micromotion. J. Shoulder Elb. Surg..

[12] Pilliar R.M., Lee J.M., Maniatopoulos C. (1986). Observations on the effect of movement on bone ingrowth into porous-surfaced implants. Clin. Orthop. Relat. Res..

[13] Harmer L., Throckmorton T., Sperling J.W. (2016). Total shoulder arthroplasty: Are the humeral components getting shorter?. Curr. Rev. Musculoskelet. Med..

[14] Kumar S., Sperling J.W., Haidukewych G.H., Cofield R.H. (2004). Periprosthetic Humeral Fractures After Shoulder Arthroplasty. J. Bone Joint Surg. Am..

[15] Nagels J., Stokdijk M., Rozing P.M. (2003). Stress shielding and bone resorption in shoulder arthroplasty. J. Shoulder Elb. Surg..

[16] Raiss P., Edwards T.B., Deutsch A., Shah A., Bruckner T., Loew M., Boileau P., Walch G. (2014). Radiographic changes around humeral components in shoulder arthroplasty. J. Bone Jt. Surg. Am..

[17] Casp A.J., Montgomery S.R.J., Cancienne J.M., Brockmeier S.F., Werner B.C. (2020). Osteoporosis and Implant-Related Complications After Anatomic and Reverse Total Shoulder Arthroplasty. J. Am. Acad. Orthop. Surg..

[18] Ritter D., Denard P.J., Raiss P., Wijdicks C.A., Bachmaier S. (2024). Preoperative 3-dimensional computed tomography bone density measures provide objective bone quality classifications for stemless anatomic total shoulder arthroplasty. J. Shoulder Elb. Surg..

[19] Ritter D., Denard P.J., Raiss P., Wijdicks C.A., Werner B.C., Bedi A., Müller P.E., Bachmaier S. (2024). Machine Learning Models Can Define Clinically Relevant Bone Density Sub-groups based on Patient Specific Calibrated CT Scans in Patients Undergoing Reverse Shoulder Arthroplasty. J. Shoulder Elb. Surg..

[20] Hayden A., Cotter E.J., Hennick T., Hetzel S., Wollaeger J., Anderson S., Grogan B.F. (2023). Bone Quality in Total Shoulder Arthroplasty: A Prospective Study Correlating CT Hounsfield Units with Thumb Test and FRAX Score. JSES Int..

[21] Martin E.J., Duquin T.R., Ehrensberger M.T. (2021). Reverse Total Shoulder Arthroplasty Baseplate Stability in Superior Bone Loss with Augmented Implant. J. Shoulder Elb. Arthroplast..

[22] Smith A.F., Frankle M.A., Cronin K.J. (2024). Maximizing Implant Stability in the Face of Glenoid Bone Stock Deficiency. Orthop. Clin. North. Am..

[23] Friedman R., Stroud N., Glattke K., Flurin P.H., Wright T., Zuckerman J., Roche C. (2015). The impact of posterior wear on reverse shoulder glenoid fixation. Bull. Hosp. Jt. Dis..

[24] Ritter D., Denard P.J., Raiss P., Wijdicks C.A., Bachmaier S. (2024). A Stemless Anatomic Shoulder Arthroplasty Design Provides Increased Cortical Medial Calcar Bone Loading in Variable Bone Densities Compared to a Short Stem Implant. JSES Int..

[25] Shah A., Werner B., Gobezie R., Denard P., Harmsen S., Brolin T., Bercik M., Thankur S., Doody S., Knopf D. (2024). Quantifying bone loss and lateralization with standardized baseplate versus augmented baseplates. JSES Int..

[26] Werner B.C., Lin A., Lenters T.R., Lutton D., Creighton R.A., Port J., Doody S., Metcalfe N., Knopf D. (2024). Influence of backside seating parameters and augmented baseplate components in virtual planning for reverse shoulder arthroplasty. J. Shoulder Elb. Surg..

[27] (2017). Standard Test Methods for Dynamic Evaluation of Glenoid Loosening or Disassociation.

[28] Colasanti C.A., Lin C.C., Ross K.A., Luthringer T., Elwell J.A., Roche C.P., Virk M.S., Simovitch R.W., Routman H.D., Zuckerman J.D. (2023). Augmented baseplates yield optimum outcomes when compared with bone graft augmentation for managing glenoid deformity during reverse total shoulder arthroplasty: A retrospective comparative study. J. Shoulder Elb. Surg..

[29] Boileau P., Morin-Salvo N., Gauci M.-O., Seeto B.L., Chalmers P.N., Holzer N., Walch G. (2017). Angled BIO-RSA (bony-increased offset–reverse shoulder arthroplasty): A solution for the management of glenoid bone loss and erosion. J. Shoulder Elb. Surg..

[30] Simcox T.G., Hao K.A., Dada O., Beason A.M., Khlopas A., Farmer K.W., King J.J., Schoch B.S., Wright T.W., Struk A.M. (2024). Survivorship and Clinical Outcomes of Reverse Total Shoulder Arthroplasty in Patients with Large Glenoid Defects Using the Stilting Technique and a Baseplate with Central Ingrowth Cage and Peripheral Locking Screws. J. Shoulder Elb. Surg..

[31] VandeKleut M.L., Yuan X., Teeter M.G., Athwal G.S. (2022). Bony increased-offset reverse shoulder arthroplasty vs. metal augments in reverse shoulder arthroplasty: A prospective, randomized clinical trial with 2-year follow-up. J. Shoulder Elb. Surg..

[32] Wittmann T., Denard P.J., Werner B.C., Raiss P. (2024). Glenoid lateralization in reverse shoulder arthroplasty: Metal vs. bone offset in different implant designs. JSES Int..

[33] Sandberg O.H., Aspenberg P. (2016). Inter-trabecular bone formation: A specific mechanism for healing of cancellous bone. Acta Orthop..

[34] Achors K., Diaz M.A., Simon P., Hill B., Christmas K.N., Cronin K.J., Frankle M.A. (2022). Avoiding Glenoid Baseplate Fixation Failure by Altering Surgical Technique for Varying Bone Densities. JBJS Open Access.

[35] Eggermont F., Verdonschot N., van der Linden Y., Tanck E. (2019). Calibration with or without phantom for fracture risk prediction in cancer patients with femoral bone metastases using CT-based finite element models. PLoS ONE.

[36] Free J., Eggermont F., Derikx L., van Leeuwen R., van der Linden Y., Jansen W., Raaijmakers E., Tanck E., Kaatee R. (2018). The effect of different CT scanners, scan parameters and scanning setup on Hounsfield units and calibrated bone density: A phantom study. Biomed. Phys. Eng. Express.

[37] Tabarestani T.Q., Levin J.M., Warren E., Boadi P., Twomey-Kozak J., Wixted C., Goltz D.E., Wickman J., Hurley E.T., Anakwenze O. (2023). Preoperative glenoid bone density is associated with systemic osteoporosis in primary shoulder arthroplasty. Semin. Arthroplast. JSES.

[38] Levin J.M., Rodriguez K., Polascik B.A., Zeng S., Warren E., Rechenmacher A., Helmkamp J., Goltz D.E., Wickman J., Klifto C.S. (2022). Simple preoperative radiographic and computed tomography measurements predict adequate bone quality for stemless total shoulder arthroplasty. J. Shoulder Elb. Surg..

[39] Cronin K.J., Vaughan A., Tzeuton S., Abboud J.A. (2023). Prospective assessment of osteoporosis in total shoulder arthroplasty. Semin. Arthroplast. JSES.

[40] Lee D.C., Hoffmann P.F., Kopperdahl D.L., Keaveny T.M. (2017). Phantomless calibration of CT scans for measurement of BMD and bone strength-Inter-operator reanalysis precision. Bone.

